# Intramolecular glycosylation

**DOI:** 10.3762/bjoc.13.201

**Published:** 2017-09-29

**Authors:** Xiao G Jia, Alexei V Demchenko

**Affiliations:** 1 Department of Chemistry and Biochemistry, University of Missouri – St. Louis, One University Blvd., 434 Benton Hall (MC27), St. Louis, MO 63121, USA

**Keywords:** carbohydrates, glycosylation, intramolecular reactions, oligosaccharides

## Abstract

Carbohydrate oligomers remain challenging targets for chemists due to the requirement for elaborate protecting and leaving group manipulations, functionalization, tedious purification, and sophisticated characterization. Achieving high stereocontrol in glycosylation reactions is arguably the major hurdle that chemists experience. This review article overviews methods for intramolecular glycosylation reactions wherein the facial stereoselectivity is achieved by tethering of the glycosyl donor and acceptor counterparts.

## Introduction

With recent advances in glycomics [1–2], we now know that half of the proteins in the human body are glycosylated [3], and cells display a multitude of glycostructures [4]. Since glycan and glycoconjugate biomarkers are present in all body fluids, they offer a fantastic opportunity for diagnostics. Changes in the level of glycans, as well as changes in glycosylation and branching patterns, can indicate the presence and progression of a disease [5–9]. With a better understanding of functions of carbohydrates, the quest for reliable synthetic methods has launched, thus elevating the priority for improving our synthetic competences. The development of new methods for stereocontrolled glycosylation [10–14] in application to the expeditious synthesis of oligosaccharides represents a vibrant worldwide effort [15–32]. Nevertheless, despite extensive studies that have emerged since the very first experiments performed by Arthur Michael and Emil Fischer in the late 1800’s, the glycosylation reaction remains challenging to chemists.

**Scheme 1:**
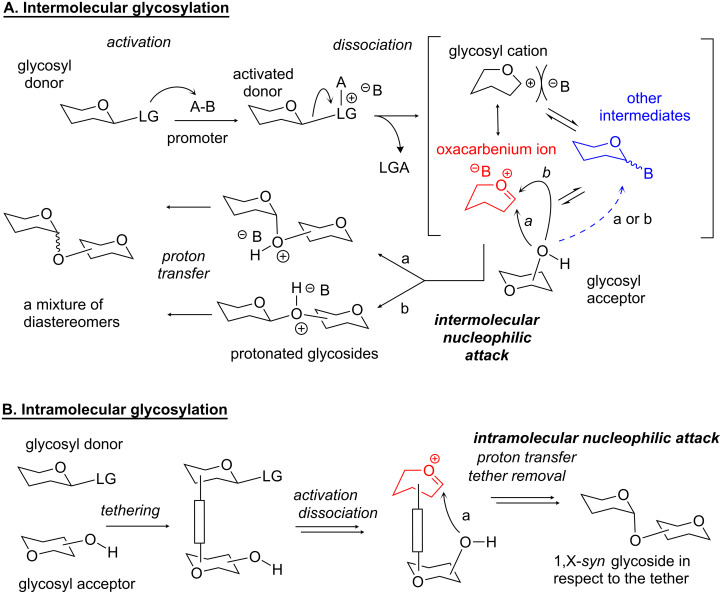
The mechanistic outline of the intermolecular (a) and intramolecular (b) glycosylation reactions.

Enzymatic glycosylation reactions are highly stereoselective [33]. However, the stereocontrol of chemical glycosylation reactions remains cumbersome despite of significant advances. Common intermolecular glycosylation reactions in the absence of a participating auxiliary typically proceed with poor stereoselectivity. In these systems, there are no forces that are able to direct the glycosyl acceptor attack on the activated glycosyl donor that exists as a flattened oxacarbenium intermediate (Scheme 1a). Early attempts to achieve some stereocontrol of glycosylations were mainly dedicated to the development of participating groups and optimization of the reaction conditions. More recently, the research emphasis is switching towards understanding of other, more fundamental factors and aspects of glycosylation. Extensive studies dedicated to conformation, configuration, stereoelectronics of the starting material, and key reaction intermediates have emerged [34–37].

Beside these attempts, an area of the intramolecular glycosylation has also been developed with an idea of providing higher efficiency of glycosylation reactions by bringing the reaction counterparts in a close proximity to each other. In many variations of this general concept, the intramolecular approach also allows for achieving better stereocontrol in comparison to that of an intermolecular reaction. This is usually credited to the facial selectivity for the glycosyl acceptor attack restricted by the tethering (Scheme 1b). However, the execution of this concept requires additional steps for the preparation of the tethered donor–acceptor combinations, and in some cases post-glycosylational modifications are also required. As a result, glycosylation that is already a four-step process (activation, dissociation, nucleophilic attack, proton transfer, Scheme 1a) has to be supplemented with additional manipulations that could lead to the decrease in over-all efficiency and yields. Hence, intramolecular glycosylations have a particular relevance to special cases of glycosylation or particularly challenging targets, such as 1,2-*cis* glycosides, where other, more direct methods fail to provide acceptable results.

**Figure 1:**
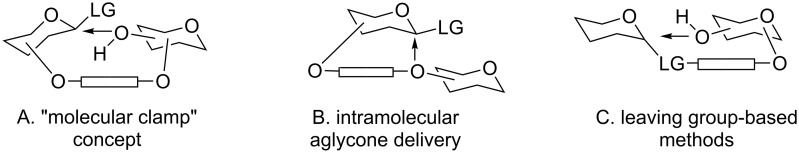
Three general concepts for intramolecular glycosylation reactions.

Presented herein is an overview of methods that have been developed to achieve higher efficiency and/or better stereoselection by tethering the donor and acceptor counterparts, reactions that are commonly referred to as intramolecular glycosylations. A number of approaches for connecting the reaction counterparts, glycosyl donor and acceptor together, have been developed to provide the enhanced facial selectivity for the acceptor attack [38–41]. Beyond early intramolecular glycosylations achieved via the orthoester rearrangement by Lindberg [42] and Kochetkov [43], as well as the decarboxylation of glycosyl carbonates by Ishido [44], Barresi and Hindsgaul [45] are often credited for the invention of the intramolecular glycosylation in 1991. However, it is a pioneering albeit less known research by Kusumoto et al. in 1986 [46] that actually started the developments in this area. Of this general idea for the intramolecular glycosylation, three different concepts have been invented: a “molecular clamp” approach, intramolecular aglycone delivery (IAD), and leaving group-based methods (approaches A–C, Figure 1). This review will discuss recent developments in the field of intramolecular glycosylations with the main emphasis on the developments of the past decade. A similar overview, albeit with the emphasis on molecular clamping, was presented as an introduction to the doctoral dissertation by Jia [47]. For previous developments in this area the reader should refer to a number of comprehensive overviews of intramolecular glycosylations in general [38–40] and IAD in particular [41,48–50].

## Review

### Molecular clamping method

#### Early developments

The “molecular clamp” concept (approach A, Figure 1) represents the first general concept for a intramolecular glycosylation strategy. The attachment of the glycosyl donor and acceptor via a tether takes place away from the reactive centers. These attachment strategies clearly distinguish the molecular clamp method from other intramolecular concepts wherein the attachment involves one of the reactive sites, acceptor hydroxy group in IAD or the leaving group of the donor. “Molecular clamping” was introduced by Kusumoto et al. [46], however, this term was coined by the same group much later [51]. We adopt this term to generally refer to this concept, which in other applications was also named “intramolecular glycosylation of prearranged glycosides” by Ziegler [52–53], “template-directed cyclo-glycosylation” by Valverde et al. [54], “remote glycosidation” by Takahashi [55] and “templated oligosaccharide synthesis” by Demchenko [56].

**Scheme 2:**
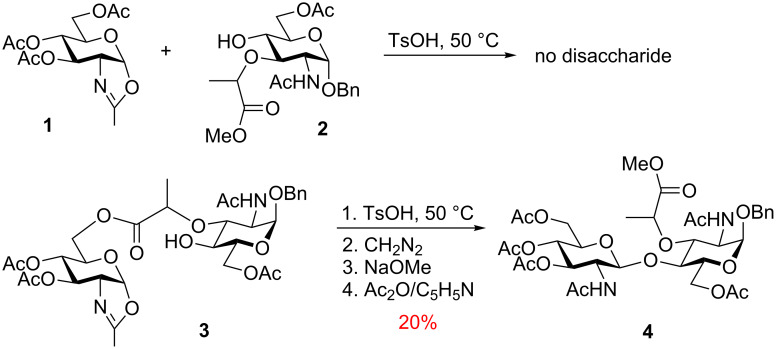
First intramolecular glycosylation using the molecular clamping.

Initially introduced by Kusumoto et al. in 1986 [46], the molecular clamping clearly demonstrated the advantages that intramolecular glycosylations can offer. The first attempt to obtain a target disaccharide quipped with muramic acid from donor **1** and acceptor **2** failed (Scheme 2). The authors rationalized that *“… a novel device was required to facilitate the coupling. We thus tried to connect the two components prior to the glycosidation reaction with an ester linkage which can be formed more readily than a glycosidic bond. ... The glycosylation reaction then becomes an intramolecular process and hence could be expected to proceed more easily.”* The authors then refer to a known phenomenon in the field of peptide chemistry *“where two components to be coupled had been brought close together by auxiliary groups.”*

With this general idea in mind, and after *“examination of molecular models”* the authors created compound **3** that was tethered via the muramic acid moiety to the C-6 position of the donor that in their opinion was *“sterically most favorable for the formation of β(1→4) glycoside.”* Indeed, after sequential glycosylation in the presence of TsOH at 50 ^o^C, methanolysis, and per-acetylation, disaccharide **4** was isolated in 20% yield. The authors then very reasonably concluded that “*Consequently, the presence of the ester linkage which kept the two sugar moieties in close proximity to each other certainly favored the formation of the desired glycoside bond in the above experiment. Thus, this is the first example of the so-called “entropic activation” in glycosidation reaction*.” The authors have also projected that the *“entropic activation demonstrated in this work seems to have wide applicability…”* and disclosed their attempts to link the reaction counterparts with dicarboxylic acids. This served as an ultimate perspective on future developments in the field, but about a decade had passed before Ziegler resurrected this concept.

#### Flexible succinoyl and related tethers

**Scheme 3:**
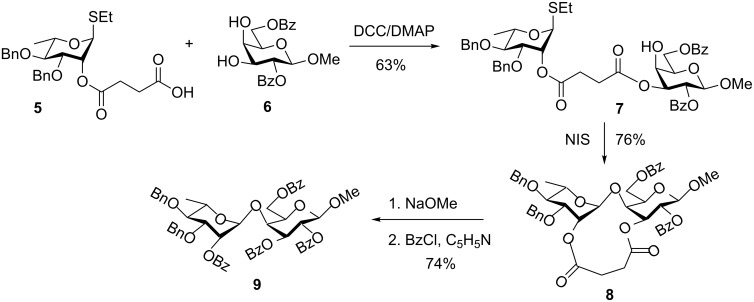
Succinoyl as a flexible linker for intramolecular glycosylation of prearranged glycosides.

Ziegler and co-workers investigated the use of a flexible succinoyl linker to link the glycosyl donor and acceptor counterpart. This reaction was named “intramolecular glycosylation of prearranged glycosides” [52–53]. Like in all “molecular clamp” applications, the tethering of the reaction counterparts takes place at positions not directly involving glycosylation sites: acceptor hydroxy group, like in the IAD or the donor leaving group, like in the leaving group-based approaches. In accordance with Ziegler’s execution of this concept shown in Scheme 3, glycosyl donor **5** equipped with the succinoyl group at C-2 was coupled to the diol galactosyl acceptor **6** in the presence of DCC and DMAP. The resulting tether compound **7** was obtained in 63% yield. The intramolecular glycosylation of the latter gave cyclic compound **8** in 76% yield, which was sequentially deacylated and per-benzoylated to afford disaccharide **9** in 74% as a pure 1,2-*trans* isomer [52]. Expansion of this approach to other positions and sugar series showed that the stereoselectivity could be relaxed, and seemed to be dependent of the donor–acceptor match–mismatch. Thus, when succinoyl was attached to the 6-OH of the galactosyl acceptor, equal amounts of α- and β-anomers were obtained. Also, when a glucosyl acceptor was employed, mainly the 1,2-*cis*-linked product was obtained.

Valverde et al. also investigated succinoyl tethers [54], but their studies were mainly focusing on phthaloyl and non-symmetrical linkers described below. Among other flexible linkers investigated are carbonate [57], as well as oxalic [57], malonic [53,57–58], and glutaric [59] dicarboxylic acids. However, like in the case of succinoyl linkers, higher flexibility led to more relaxed stereoselectivity. Further variations upon this method involved the modification of the macrocycle ring size, torsional rigidity of the spacer, position of the attachment to both donor and acceptor, relative configuration of hydroxy groups, and the length of the linker [58,60–72]. Among early examples, xylylene and phthalimido linker showed very high efficiency, and will be highlighted below. Another early development discussed below is the peptide-templated synthesis. Beyond these influential early studies that led to further developments, this topic was comprehensively overviewed and for early developments the reader should refer to the original references and excellent comprehensive overviews of the topic [38,40]. It is a commonly accepted fact that the outcome of many glycosylations that fall under the general molecular clamp concept can be unpredictable. Therefore, practically every approach developed under this category was extensively studied and applied to a variety of sugar series and targets [58,73–74].

#### Phthaloyl and related tethers

**Scheme 4:**
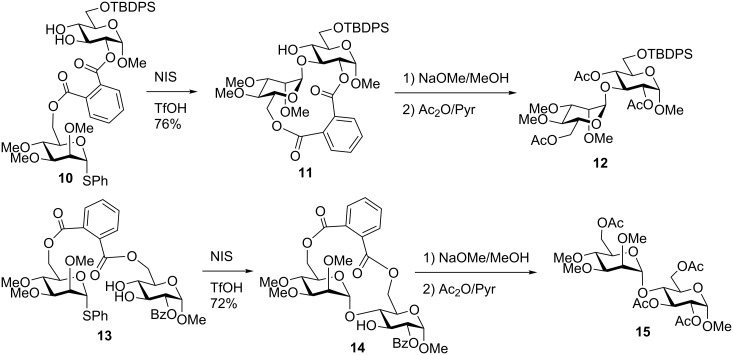
Template-directed cyclo-glycosylation using a phthaloyl linker.

Phthaloyl tethering was also introduced by Ziegler [53] and practically concomitantly by Valverde et al. [54] as “template-directed cyclo-glycosylation.” In the latter application, glycosyl donor precursors were reacted with phthalic anhydride to afford the corresponding esters. The activation with thionyl chloride was used for tethering the donors to the glycosyl acceptor counterpart and the regioselectivity was controlled using tin-mediated coupling under microwave irradiation. The tethered compound **10** was then glycosylated in the presence of NIS/TfOH to afford compound **11** (Scheme 4). The tether was removed with NaOMe and the product was globally acetylated to afford **12** as an α-(1→3)-linked isomer. The regioselectivity in this case was driven by the phthaloyl tether attachment to the neighboring C-2 position. In contrast, 6,6’-linked donor–acceptor pair **13** led to the formation of the (1→4)-linked regioisomer **15** [64]. Apparently, the rigid phthaloyl tether helps to achieve high regioselectivity because the anomeric center of the activated donor cannot easily reach out for hydroxy groups at remote positions.

In other applications, such as in the glucosyl donor series, this application was less effective. For instance, relaxed regioselectivity was observed in cases when the phthaloyl linker was attached to the primary position of the acceptor [64]. Also, relaxed stereoselectivity was observed in case of glucosyl donors equipped with a non-participating group at C-2. Valverde at al. also investigated isophthalic tether, derived from benzene-1,3-dicarboxylic acid, and observed improved stereoselectivity in a number of applications [65]. The phthalimido tethering was further extended to a number of useful applications including the synthesis of branched structures by Takahashi and cyclodextrins by Fukase discussed below.

**Scheme 5:**
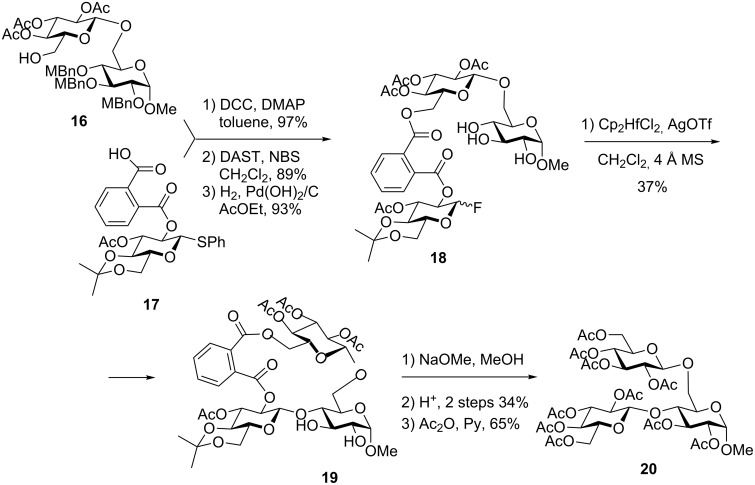
Phthaloyl linker-mediated synthesis of branched oligosaccharides via remote glycosidation.

Thus, Takahashi et al. considered both flexible succinoyl and the rigid phthaloyl tether, but based on the outcome of the computational studies of relative conformations and energies chose the latter linker [55]. To apply the remote glycosidation methodology to the synthesis of the 4,6-branched trisaccharide, phthaloylated thioglycoside **17** was coupled with the 6-hydroxy group of the acceptor precursor **16** in the presence of DCC and DMAP (Scheme 5). The tethering was accomplished in 97% yield and the resulting conjugate was converted into glycosyl fluoride by the treatment with DAST and NBS in 89% yield. Finally, selective cleavage of *p*-methylbenzyl ethers was accomplished with H_2_ over Pd(OH)_2_/C to provide donor–accepter conjugate **18** in 93% yield. Subsequent remote glycosidation of **18** was conducted in the presence of Cp_2_HfC1_2_ and AgOTf in CH_2_C1_2_ under reflux. The cyclized product **19** was obtained in 37% yield, the tether was removed with NaOMe, and the resulting free hydroxy groups were acetylated to afford the branched trisaccharide **20**.

**Scheme 6:**
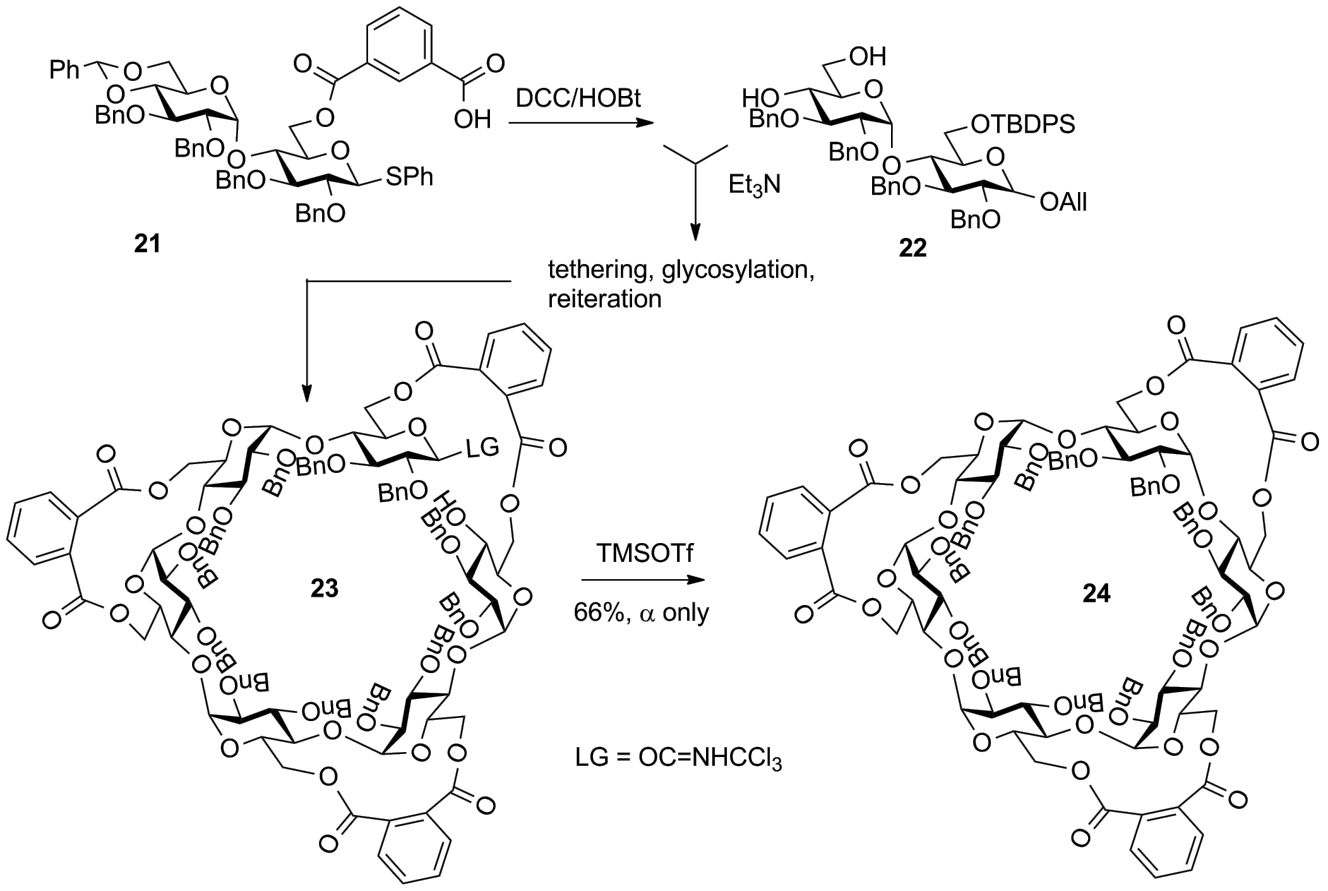
Molecular clamping with the phthaloyl linker in the synthesis of α-cyclodextrin.

The chemical synthesis of cyclodextrins is very challenging: controlling α-gluco stereoselectivity, and especially the final cyclization, represent a great challenge. For example, in Ogawa’s synthesis of α-cyclodextrin the chain assembly was non-stereoselective and the cyclization was achieved in only 21% yield [75]. Kusumoto et al. clearly demonstrated the advantage of the molecular clamping in application to the synthesis of α-cyclodextrin (Scheme 6) [51]. The tethering was used to improve the selectivity during the stepwise chain elongation via the coupling of maltose building blocks **21** and **22**, as well as the efficiency of macrocyclization. The macrolactonization using the phthaloyl group clamp was accomplished using DCC and DMAP in refluxing 1,2-dichloroethane. A fairly high dilution (0.04 M) allowed to achieve the formation of the cyclic ester in 79% yield. This impressive yield was explained by the ability of the phthaloyl clamping groups to present the oligosaccharide chain in a favorable conformation for cyclization. After hydrolyzing the anomeric protecting group, several conditions were tried to close the ring and glycosylation with the trichloroacetimidoyl leaving group in **23** activated with trimethylsilyl triflate gave the desired α-linked product **24** in 66% yield [51].

#### Xylylene tether

**Scheme 7:**
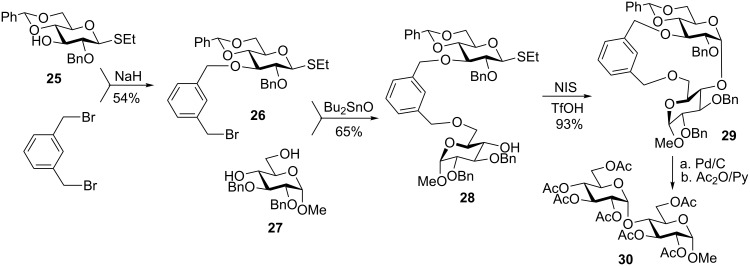
*m*-Xylylene as a rigid tether for intramolecular glycosylation.

Generally during glycosylation, it has been found that the more rigid the spacers, and smaller macrocycle formed, the more selective the reaction [63,69]. As an example of this approach, a rigid xylylene linker introduced by Schmidt [68], was successfully applied to the intramolecular synthesis of 1,2-*cis* glycosides with complete selectivity (Scheme 7) [69]. Thus, thioglycoside **25** is first alkylated at C-3 position. The resulting intermediate **26** is then used as the alkylating reagent to create a tether to acceptor **27** using tin-mediated primary alkylation to afford the tethered pair **28**.

The latter is then intramolecularly glycosylated in the presence of NIS/TfOH in 93% yield and complete stereoselectivity. The resulting cyclic compound **29** is then subjected to concomitant xylylene tether removal and debenzylation followed by global acetylation to afford product **30**.

**Scheme 8:**
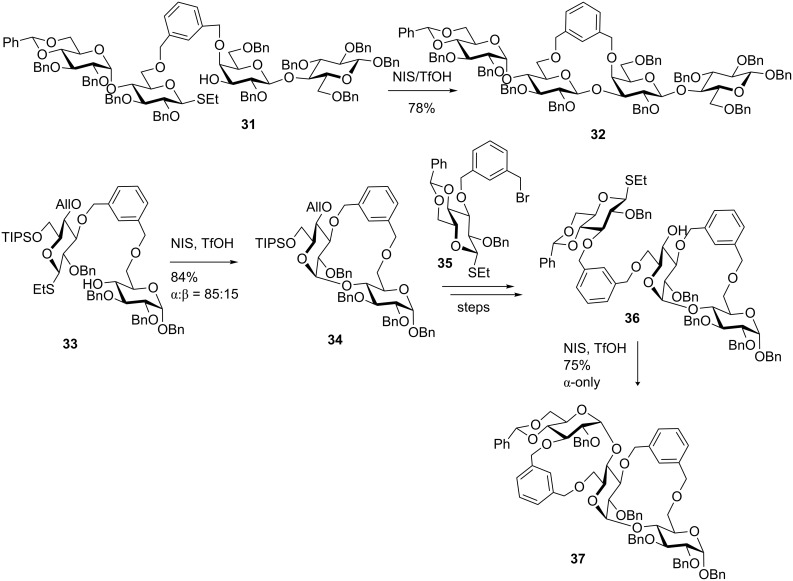
Oligosaccharide synthesis using rigid xylylene linkers.

The extension of this approach to convergent oligosaccharide synthesis and reiterative sequencing in presented in Scheme 8. Thus, maltose and lactose disaccharide building blocks were linked via the xylylene tether, and the resulting compound **31** was glycosylated in the presence of NIS/TfOH to afford tetrasaccharide **32** in 78% as a pure β-diastereomer [70]. Schmidt demonstrated the usefulness of xylylene tethers in application to the iterative synthesis of maltotriose [70]. In this application, the xylylene tether was used to link two glucose derivatives via the 3’- and the 6-positions to create a tethered combination **33** (Scheme 8). NIS/TfOH was then applied to glycosylate the two sugar units to give disaccharide **34** in 84% yield (α/β = 85:15). Subsequent selective deprotection of the 6’-position, introduction of the new donor moiety **35** followed by liberating the hydroxy group at C-4’ gave the tethered donor–acceptor combination **36**. After the NIS/TfOH-promoted glycosylation the desired trisaccharide **37** was obtained in 75% yield as a pure α-linked diastereomer. The per-acetylated maltotriose target was obtained after palladium-catalyzed hydrogenation that affected the removal of the template and all benzyl protecting groups followed by acetylation of the resulting hydroxy groups.

#### Peptide tether/template

**Scheme 9:**
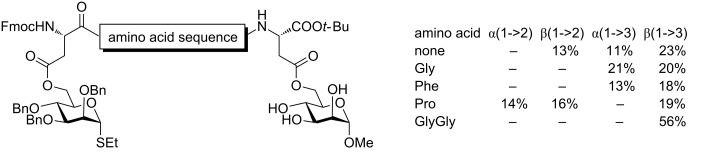
Stereo- and regiochemical outcome of peptide-based linkers.

Short peptide chains have also been investigated as templates for glycosylation. The general underpinning idea is to streamline the oligosaccharide synthesis and purification by using well developed peptide coupling reactions with or without the use of solid phase methods. To execute this concept, Fairbanks et al. investigated a number of peptide chains with various amino acids as templates (Scheme 9) [76–77]. Using DCC-mediated coupling reactions asparagine was attached both to a mannose donor and a trihydroxymannose acceptor, and the central amino acid unit(s) was varied. Intramolecular glycosylation was carried out with NIS/TfOH, resulting in a mixture of disaccharide products showing slight regioselectivity bias towards the formation of (1→3) linkages.

**Scheme 10:**
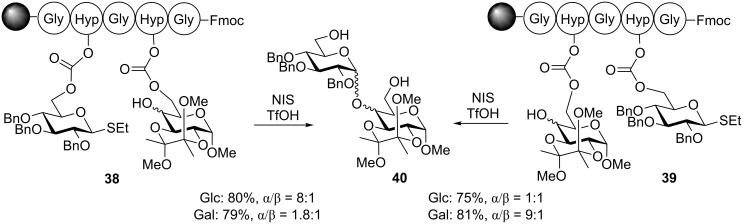
Positioning effect of donor and acceptor in peptide templated synthesis.

The stereoselectivity of these linkages can vary, but it was typically very relaxed perhaps due to a fairly low rigidity of this type of a template. Hence, further development of this methodology focused on solid-supported peptide templates [78]. For instance, Warriner and co-workers investigated a solid supported peptide sequence that was connected to the 6-hydroxy groups of the sugar units using carbonate linkages (Scheme 10) [79]. The hydroxyproline (Hyp, (2*S*,4*R*)-4-hydroxypyrrolidine-2-carboxylic acid) linked glycosyl donor and acceptor system failed to provide the product of the intramolecular glycosylation, probably due to steric interactions. A glycine residue spacer was found necessary to separate the two rigid Hyp bound counterparts. Thus, glycosylation of conjugate **38** in the presence of NIS and TMSOTf resulted in the formation of the (1→4)-linked disaccharide **40** in 80% yield with high α-selectivity (α/β = 8:1). Interestingly, when the donor and acceptor positions on the peptide were reversed, such as conjugate **39**, glycosidation of this compound produced disaccharide **40** in 75% yield albeit the stereoselectivity was entirely lost (α/β = 1:1). Galactosyl acceptors also showed a dramatic effect of the relative position of the donor and acceptor on the peptide sequence. Intriguingly, the stereoselectivity outcome was reversed (1.8:1 and 9:1) in comparison to glucosyl acceptors. When a similar concept was applied to mannosyl acceptor low 2:1 stereoselectivity was obtained regardless of the relative positioning of the reaction counterparts. This peptide-based templating was extended to the synthesis of a small library of disaccharides.

#### Non-symmetrical and other tethers

**Scheme 11:**
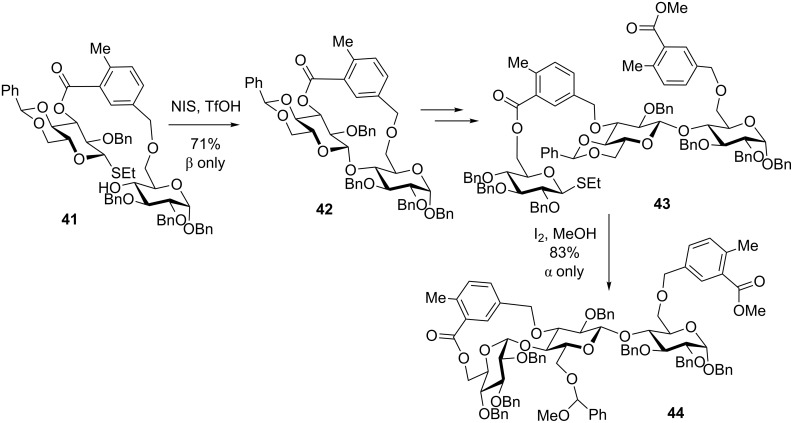
Synthesis of a trisaccharide using a non-symmetrical tether strategy.

Non-symmetrical templates have also been developed with a general idea of achieving differentially cleavable attachments that could provide more flexibility in the synthesis of longer oligosaccharides [62,65]. Some representative examples of this general concept include benzyl–silicon tether [72], which is a hybrid approach to xylylene and a regular silicon [59] type of tethering. Another example of a non-symmetrical tethering strategy is benzyl–benzoyl hybrid tethering [72] that elaborated on xylylene and phthaloyl tethering approaches discussed above. Thus, this strategy was used in the synthesis of a trisaccharide through reiterative template-assisted synthesis (Scheme 11). Compound **41**, wherein the donor and acceptor counterparts were subjected to tethering via this rigid hybrid linker, was subjected to the NIS/TfOH-promoted glycosylation. The tether in the resulting disaccharide **42** could then be selectively opened with NaOMe. This leads to liberating only one hydroxy group (at C-3”) that could be used for tethering with a glycosyl donor using a similar tethering concept to afford compound **43**.

The second glycosylation reaction is conducted in the presence of iodine in methanol. These conditions allow to cleave benzylidene groups concomitantly with the activation of the leaving group. As a result, the formation of the 14-membered ring is observed and compound **44** obtained in 83% yield with complete α-stereoselectivity. The ester part of the template is then cleaved with sodium methoxide in methanol revealing the 6”-hydroxy group that can be used for subsequent transformations [72].

**Scheme 12:**
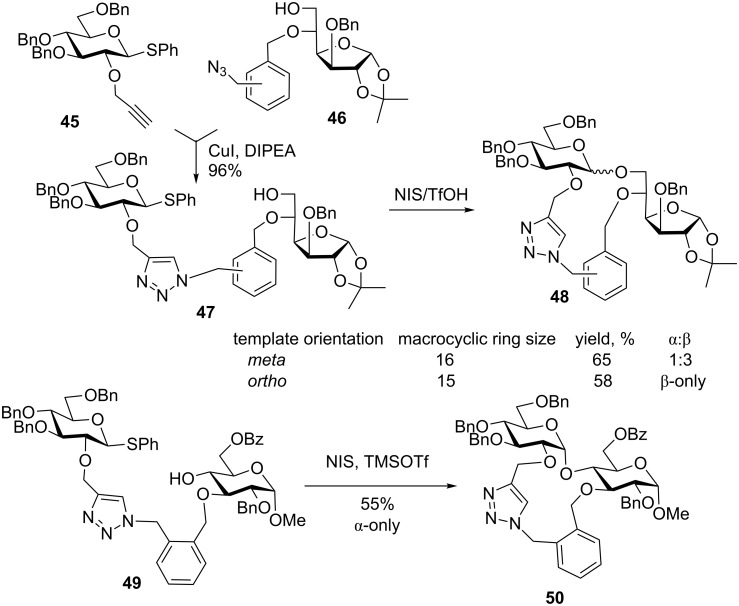
Effect of ring on glycosylation with a furanose.

In a recent attempt to simplify the synthesis of the non-symmetrical tethers, a highly trendy triazole-forming click chemistry was combined with rigid spacers by the Schmidt group. α,α’-Dibromo *ortho*- and *meta*-xylene-derived rigid spacers were used in this application, and this approach allowed to investigate the size of the macrocycle formed during the glycosylation (Scheme 12) [80–81]. Thioglycoside donor **45** containing a 2-*O*-propargyl group and acceptor **46** with an azide-containing protecting group were connected using a click reaction to afford the tethered intermediate **47**. Upon treatment with NIS/TfOH, disaccharide **48** was obtained with complete β-selectivity when the *ortho*-xylyl group (15-membered ring) was used, versus α/β = 1:3 selectivity in the case of the *meta*-xylene linked counterpart [80]. As in the previous example with the xylylene-derived linker, the triazole linker was removed under standard hydrogenation conditions followed by global acetylation. The results obtained with the 6-hydroxyglucopyranosyl acceptor were somewhat mixed [81]. Attaching the template at various positions of the acceptor to achieve either 16- or 17-membered macrocycles resulted in high yields of 90% and 82%, respectively. However, the stereoselectivity of the reactions was modest, α/β = 3:1 and 1:2, respectively.

With the observation that selectivity can be influenced by the size of the macrocycle formed as a result of the intramolecular glycosylation, a tethered system linked via the O-3 position with the acceptor **49** was obtained (Scheme 12). Following the NIS/TMSOTf-promoted glycosylation, macrocycle **50** was formed in 55% yield with exclusive α-stereoselectivity. Interestingly, when a similar template was attached to the O-2 position followed by glycosylation with the 3-hydroxy group, the reaction proceeded with high β-selectivity. With the varying anomeric stereoselectivities and yields, it was hypothesized that the benzylic methylene group may be responsible for the increased rotational freedom between the triazoyl and benzyl moieties. Investigations with *o*-azidobenzyl protecting groups were used to reduce the degrees of freedom and also to form smaller ring sizes [81].

#### Templated oligosaccharide synthesis

**Scheme 13:**
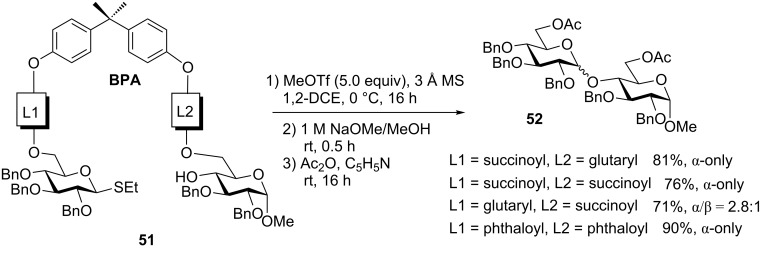
Rigid BPA template with various linkers.

Recently, Demchenko and co-workers introduced templated oligosaccharide synthesis, wherein bisphenol A (BPA) was used as the template and succinoyl, glutaryl or phthaloyl linkers were used to tether glycosyl donors and acceptors together [56,82]. The templated synthesis also falls into the general molecular clamping method. High stereoselectivity could be achieved with both flexible and rigid linkers (L1 and L2, Scheme 13). However, the use of the rigid BPA template core appears to be the key to ensure the high stereoselectivity because with flexible peptide core, no difference in stereoselectivity was detected. Thus, if linker L1 is shorter than L2, succinoyl vs glutaryl, respectively (or the same length, succinoyl) in compound **51**, the glycosyl acceptor counterpart is delivered from the bottom face of the activated donor. These reactions produced the corresponding disaccharide **52** in 76–81% yields and complete α-stereoselectivity. Conversely, if linker L1 is longer than L2, glutaryl vs succinoyl, respectively, the stereoselectivity is lost (α/β = 2.8:1). Interestingly, the template effect is stronger than that of a participating solvent acetonitrile that was unable to favor β-anomers, like in intramolecular glycosylations. Instead, complete β-selectivity could be achieved using glycosyl donors equipped with the participating group at C-2.

**Scheme 14:**
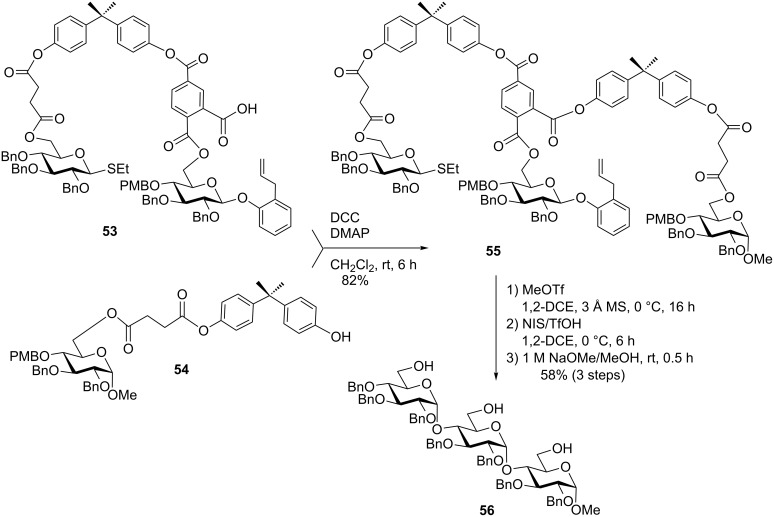
The templated synthesis of maltotriose in complete stereoselectivity.

A further mechanistic study of this work led to the appreciation of phthaloyl linkers leading to better yields, albeit complete α-selectivity [82]. To demonstrate the utility of the method a trisaccharide was synthesized using trimellitic anhydride as a precursor for the bridging linker (Scheme 14) [56]. The more flexible succinoyl linkers showed a clear advantage over more rigid phthaloyl linkers in terms of stereoselectivity and yields. Thus, a tethered donor-central unit conjugate **53** was coupled with the BPA-conjugated glycosyl acceptor **54** using DCC/DMAP-mediated coupling reaction to obtain the templated conjugate of three monosaccharide units **55** in 82% yield. The selective activation of the *S*-ethyl leaving group in compound **55** was achieved with MeOTf and the glycosylation of the central building block took place with concomitant removal of the *p*-methoxybenzyl (PMB) group. The *o*-allylphenyl leaving group was activated with NIS/TfOH, and again the PMB group of the acceptor was removed during the glycosylation step. The resulting maltotriose **56** was then released from the template by reaction with NaOMe in MeOH [56].

### Intramolecular aglycone delivery (IAD)

**Scheme 15:**
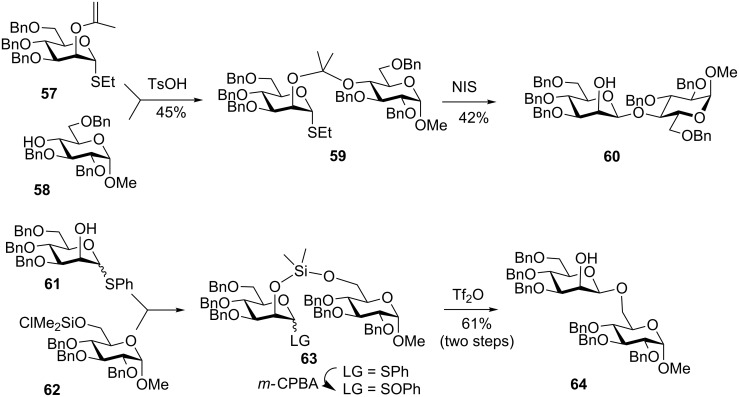
First examples of the IAD.

This approach was invented by Barresi and Hindsgaul [45] who named it intramolecular aglycone delivery (aglycon in the original literature) and it is commonly abbreviated as IAD (approach B, Figure 1). The distinctive characteristic of the IAD methods, and its major difference from other intramolecular approaches is the glycosyl donor which is tethered directly via the hydroxy group of the glycosyl acceptor to be glycosylated. In all other approaches, the acceptor is linked away from the hydroxy group that is to be glycosylated. The tethering site at the glycosyl donor can be either the neighboring C-2 position or a remote position. Barresi and Hindsgaul employed the activation of the thioethyl leaving group with *N*-iodosuccinimide, which resulted in excellent stereoselectivity for the synthesis of challenging β-mannoside [45,83]. Overall, this is a two-step process: first, formation of the intermolecular ketal between the donor and acceptor counterpart, and then glycosylation directly on the ketal oxygen of the glycosyl acceptor is performed. This was accomplished by the treatment of 2-isopropenylmannose **57** in the presence of TsOH (Scheme 15) to obtain mixed ketal **59**. The second step involved glycosidation in the presence of NIS that produced disaccharide **60** in 42% yield and complete β-selectivity. Despite fair yields during both the ketal formation and glycosylation stage, this excellent idea gave rise to the development of procedures that helped to evolve the IAD method into a very effective methodology. In particular, the implementation of silyl, allyl, and more recently, naphthylmethyl tethers helped to achieve significantly higher yields in comparison to those reported in the original protocols. Since the IAD has been overviewed multiple times [41,48–50], presented herein are only the basics as well as the key recent developments of this.

Stork and Bols independently demonstrated that silicon bridge-mediated aglycone delivery helps to enhance the yields while maintaining excellent stereocontrol [84–85]. For example, the Stork group used chlorodimethylsilyl protected acceptor precursor **62** for conjugation to the 2-hydroxy group of donor **61** as shown in Scheme 15. The thiophenyl leaving group of the tethered compound **63** was then oxidized into the corresponding sulfoxide with *m*-CPBA. The latter was glycosidated in the presence of Tf_2_O to afford disaccharide **64** in complete stereoselectivity and a good yield of 61% over two steps (73% from the sulfoxide intermediate). This dimethylsilyl linker strategy was also applied towards the synthesis of α-glucosides by Bols [85].

Subsequently, the Bols group expanded the scope of the IAD method by investigating long-range tethering [39,85–89]. In this application the tether attachment was placed away from the anomeric center offering a more flexible mode for obtaining either 1,2-*cis* or 1,2-*trans* linkages depending on the placement of the tether. While complete stereoselectivities were obtained with a ribofuranosyl donor tethered at C-5, application of the long range IAD towards glucopyranosides was less successful. Among a variety of attachment points, only tethering from the C-4 position showed some promise favoring the formation of the 1,4-*syn* products. Unfortunately, the IAD from the C-3 position afforded a mixture of diastereomeric glycosides, whereas tethering from the C-6 position gave predominantly the 1,6-anhydro product.

**Scheme 16:**
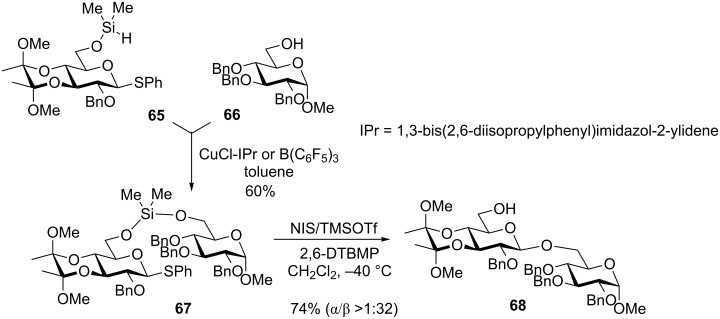
Long range IAD via dimethylsilane.

Following upon the early studies by Stork and Bols, Montgomery et al. further expanded the idea of the long range IAD via silicon tethering [90]. In the most recent report, they hypothesized that the conformational restriction of the pyranose should position the C-6 oxygen of the donor away from the developing oxacarbenium intermediate, thereby circumventing the formation of the cyclized product [91]. This was achieved by protecting the 3,4-*trans*-diol with a cyclic bis-ketal. Primary aliphatic alcohols underwent glycosylation very readily with donor **65** affording glycosides in excellent yields with high β-selectivity (>1:32). With primary glycosyl acceptors, such as **66** (Scheme 16), yields were slightly diminished due to the formation of the homocoupling products. Secondary alcohol acceptors were even less efficient showing a high substrate specificity of this approach. Other donor series including 2-azido and 2-deoxy sugars were investigated and provided similar results. This method was also applied towards the delivery of acceptors from the neighboring C-2 position [91]. This approach tolerated a much wider range of acceptors and showed excellent stereoselectivity with secondary acceptors providing high yields and complete stereoselectivities: α- for glucosides and β- for mannosides.

**Scheme 17:**
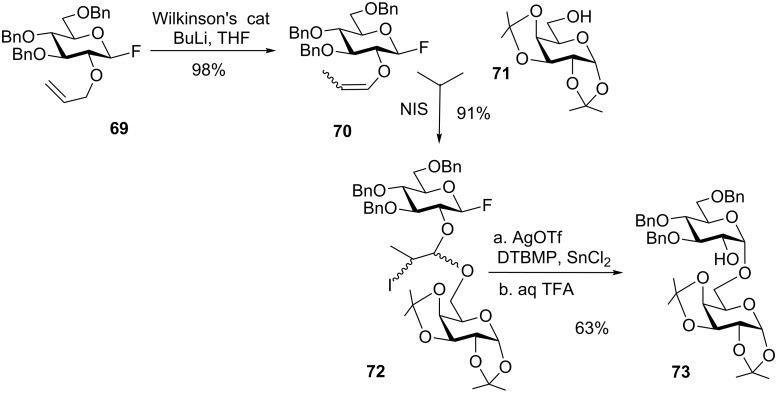
Allyl-mediated tethering strategy in the IAD.

Another direction in the development of the IAD method emerged with the introduction of the allyl-mediated strategy by Fairbanks and co-workers who achieved improved yields and complete stereoselectivity in α-glucosylations and β-mannosylations [92]. In accordance with the linking strategy, the vinyl ether **70** was obtained in 98% yield from the corresponding 2-*O*-allyl ether **69** by the treatment with Wilkinson’s catalyst and BuLi (Scheme 17) [93]. Subsequent NIS-mediated tethering of **70** and acceptor **71** gave the tethered donor–acceptor pair **72**. The latter was then intramolecularly glycosylated in the presence of silver triflate, tin(II) chloride, and 2,6-di-*tert*-butyl-4-methylpyridine (DTBMP). Finally, the tether was cleaved off using TFA to give pure 1,2-*cis* glycoside **73** in 63% yield over two steps.

An alternative linker was developed by Ito and Ogawa who implemented DDQ-mediated oxidative transformation of the *p*-methoxybenzyl (PMB) protecting group at the C-2 position of the donor into a tethering mixed acetal with a hydroxy group of the acceptor [94]. The early studies have successfully applied this PMB-based IAD method to the synthesis of a variety of oligosaccharides and glycoconjugates containing challenging β-mannosides [95–96]. A very impressive application of the IAD in polymer-supported reactions has also emerged [97]. Interestingly, the PMB tether was although used as the linker for the attachment to the polymer support. Bertozzi et al. investigated a similar concept based on 3,4-dimethoxybenzylidene tethering that was found superior in application to the synthesis of α,α-linked trehalose derivatives [98–99].

**Scheme 18:**
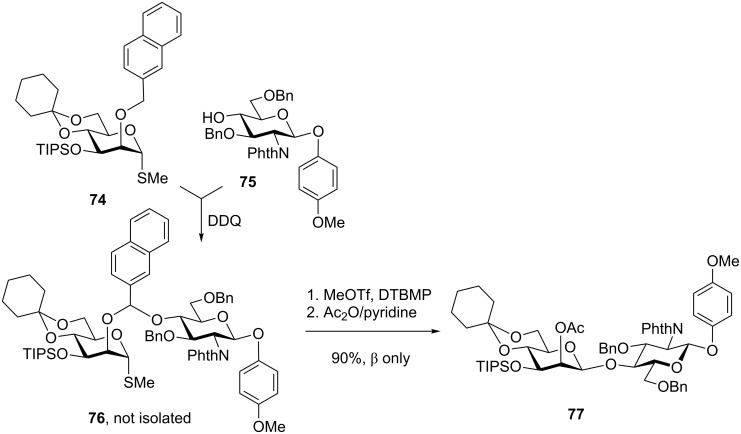
IAD using tethering via the 2-naphthylmethyl group.

A major improvement of this approach has emerged with the implementation of a 2-napthylmethyl group as a tether group into this strategy [100]. This adjustment has allowed a greater range of hindered glycosyl acceptors to be tethered and glycosylated in high yields and stereoselectivity. The versatility of this approach lies in that it generally provides significantly higher yields in comparison to practically all previously developed IAD approaches. A representative example depicted in Scheme 18 shows the synthesis of disaccharide **77**, which clearly demonstrates that in terms of the over-all yields. This approach can even compete with direct intermolecular glycosylations while providing excellent stereoselectivity. Thus, mixed acetal **76** can be readily formed in 2 h by the addition of DDQ to a mixture of donor **74** and acceptor **75**. Without further purification, the latter mixture can be glycosylated in the presence of MeOTf and DTBMP followed by acetylation to give disaccharide **77** in an excellent yield of 90% and complete β-selectivity [100]. Initially investigated for the synthesis of β-mannosides, α-glucosides, and β-arabinofuranosides [100], this approach was extended to the synthesis of β-rhamnosides [101] and many other challenging linkages and targets [41,102–108].

**Scheme 19:**
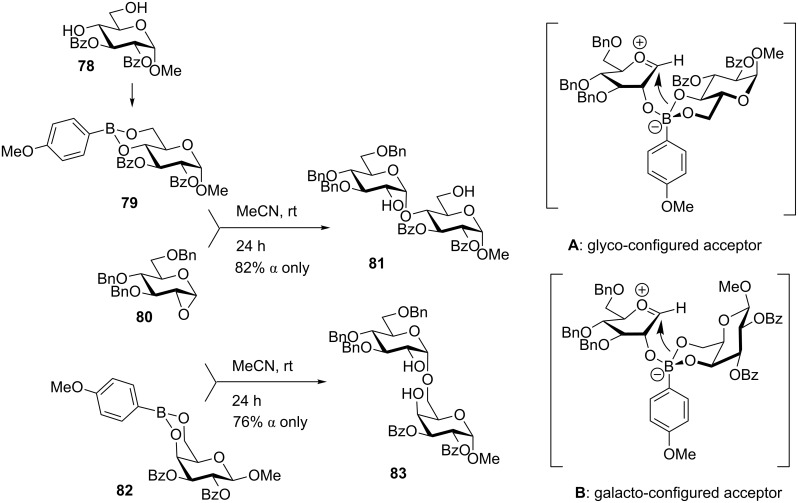
Origin of selectivity in boronic ester mediated IAD.

Arylboronic esters have recently been probed by Toshima and co-workers as a successful linkage for the IAD method [109]. The arylboronic sugar derivatives, such as **79**, can be easily obtained from the corresponding 4,6-diol **78** and a arylboronic acid in toluene at reflux (Scheme 19). Boronic ester **79** was then reacted with 1,2-anhydro donor **80**. It was assumed that this reaction proceeds via the oxacarbenium ion tethered to a tetracoordinated boronate ester. The subsequent glycosylation then proceeds regioselectively from the less-hindered boron–oxygen bond (see intermediate **A**). In this case, where gluco-configured acceptor **78** was used the (1→4)-linked product **81** was formed exclusively in 82% yield with high α-selectivity. Similarly, when mannose, glucosamine, and glucal were used as glycosyl acceptors, the 1→4 linkage was formed exclusively with high α-selectivity in 92%, 77%, and 72% yield, respectively. Conversely, the galacto-configured boronic ester acceptor **82** was used, the α-(1→6)-linked product **83** was formed in 70% yield. Again, the regioselectivity of glycosylation is driven by the less-hindered boron–oxygen bond, which is from C-6 face in the case of galactose (intermediate **B,** Scheme 19). In the case of other acceptors: a 3,4-diol of galactose gave the α-(1→4) linkage predominantly (65%) while a 2,3-diol of mannoside led to the α-(1→3)-linked disaccharide in 70% yield.

**Scheme 20:**
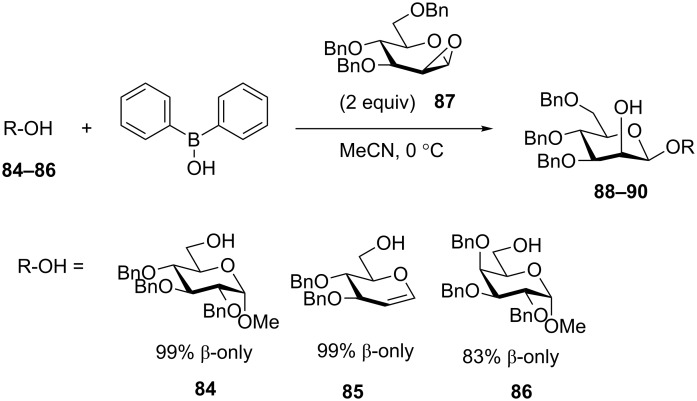
Arylborinic acid approach to the synthesis of β-mannosides.

This method has recently found a valuable extension to the synthesis of β-mannosides [110]. Thus, diphenylborinic acid-derived glycosyl acceptors **84–86** were reacted with 1,2-anhydromannosyl donor **87** (Scheme 20). The tethered oxacarbenium ion intermediate then directs the nucleophilic attack intramolecularly to the β-face of the mannosyl donor. As a result, disaccharides **88–90** were obtained in 83–99% yields and exclusive β-manno stereoselectivity. Advantages of this methodology have been tested in application to the synthesis of a tetrasaccharide repeating unit of lipopolysaccharide derived from *E. coli* O75 [111].

**Figure 2:**
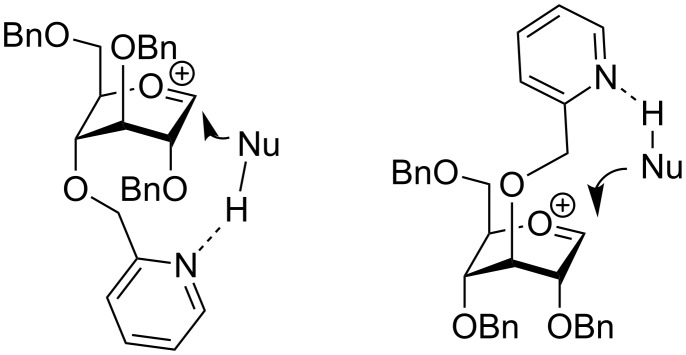
Facial selectivity during HAD.

Demchenko and co-workers introduced the use of the picolinyl group at the neighboring C-2 position of glycosyl donors as an arming participating group [112–113]. These glycosylations provided complete 1,2-*trans* stereoselectivity, *anti* with respect to the orientation of the picolinyl group. When the picolinyl ether or picoloyl ester group was placed at remote positions, glycosylations occurred *syn* with respect to the orientation of the picolinyl/picoloyl group [114]. The stereoselectivity was explained by the occurrence of the hydrogen bonding between the hydroxy group of glycosyl acceptor (NuH) and the nitrogen atom of the picolinyl/picoloyl group. Subsequently, the glycosyl acceptor is delivered towards the oxacarbenium ion from the same face (*syn*) as the picolinyl/picoloyl group (Figure 2). This method, named H-bond-mediated aglycone delivery (HAD), has been applied towards the synthesis of α-glucosides [114–116], α-galactosides, β-rhamnosides [114], and β-mannosides [117]. The latter approach was extended to the synthesis of a β-manno-trisaccharide, wherein complete β-manno selectivity was obtained at room temperature [117]. A useful extension of this method to glycosyl donors with switchable selectivity has also been disclosed by the Demchenko group [118–119].

**Scheme 21:**
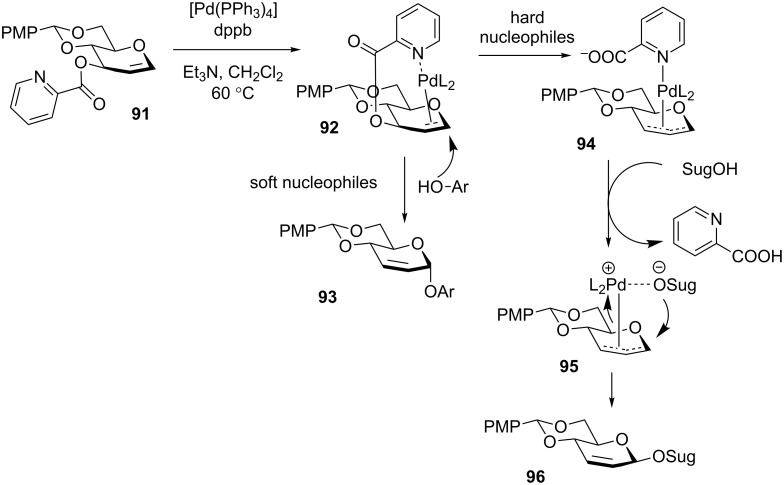
Possible mechanisms to explain α and β selectivity in palladium mediated IAD.

Not being strictly intramolecular, the HAD method led to a variety of other delivery methods, which included the realm of organometallics. For instance, Liu and co-workers have developed the use of catalytic palladium to control the stereoselectivity in glycosylations via a palladium π-allyl intermediate. Previously, the application of this technique to glycosidic bond formation has been hampered by the difficulty in the formation of the palladium π-allyl intermediates and their poor reactivity in the electron-rich glycal systems [120]. To overcome this challenge the Liu group explored the application of palladium π-allyl intermediates to *O*-glycosylation through the use of a picoloyl group to direct palladium binding at the C-3 position [121]. Glycosylation results are indicative of two reaction pathways with differing in the selectivity outcome based on the hard/soft properties of the nucleophiles. In both pathways, the first step involves picoloyl group-directed coordination of palladium from the top β-face of the 1,2-dehydro donor **91** to form intermediate **92** (Scheme 21). With softer nucleophiles, such as phenol (ArOH), the nucleophilic attack is directed away from the steric bulk of the palladium to give α-glycosides **93**. When the acceptor is a hard nucleophile, such as a sugar alcohol (SugOH), the picoloyl group is displaced to generate the π-allyl complex **94**. The harder nucleophiles then tend to coordinate to palladium via intermediate **95**, followed by intramolecular nucleophilic delivery to form β-anomer **96**. Both primary and secondary sugar acceptors worked well providing disaccharides with high β-selectivity and good yields. Overall, compounds **93** and **96**, obtained as a result of this interesting reaction, represent products of the Ferrier rearrangement, 2,3-dehydro derivatives.

### Leaving group-based methods

This overview continues with the discussion of the leaving group-based tethering concept (approach C, Figure 1). As the name of the concept implies, the glycosyl acceptor is linked (away from the glycosylation site) to the leaving group of the glycosyl donor. The first examples of this type of intramolecular glycosylation was based on the 1,2-orthoester rearrangement by Lindberg [42] and Kochetkov [43], as well as the decarboxylation of glycosyl carbonates by Ishido [44]. Intramolecular glycosylations where the glycosyl acceptor was purposefully attached directly to the leaving group of the glycosyl donor have been introduced by the Schmidt group [122]. The applicability of these techniques is still relatively unexplored, yet, it has been proposed that these reactions tend to be intermolecular rather than intramolecular [123–124]. Subsequent studies involved the exploration of various reaction conditions [125–126], and the investigation of other leaving groups [123–124,127].

**Scheme 22:**
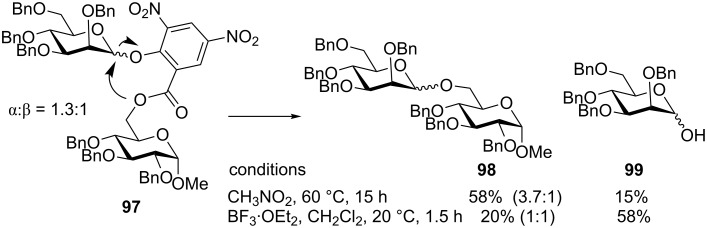
DISAL as the leaving group that favors the intramolecular glycosylation pathway.

For instance, Jensen et al. developed the methyl 3,5-dinitrosalicylate (DISAL) anomeric leaving group that could be used as a platform for linking the glycosyl acceptor in place of the methyl ester [128]. Glycosylation of conjugate **97** wherein glycosyl acceptor was linked via an ester bond at the *ortho*-position of the DISAL leaving group of the donor gave best results under elevated temperatures. Thus, mannoside **98** was obtained in 58% yield with modest stereoselectivity (Scheme 22). The yields are hampered by the competing formation of the hemiacetal product **99**. Crossover experiments with 1,2:5,6-di-*O*-isopropylidene-α-D-glucofuranose acceptor showed only disaccharides resulting from the intramolecular glycosylation. However, when crossover experiments with cyclohexanol were conducted, the intermolecularly formed cyclohexyl glycoside was found to be the major product (5.2 to 1) compared to the intramolecular glycosylation product. The addition of Lewis acids helps to reduce the reaction time and the temperature required, but also increases the formation of hydrolysis products and reduces overall stereoselectivity.

**Scheme 23:**
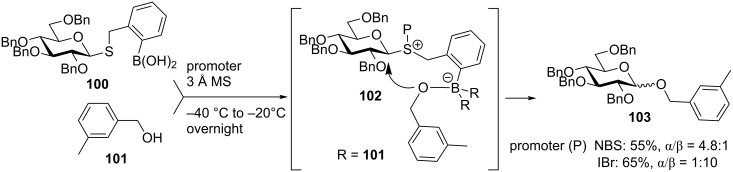
Boronic acid as a directing group in the leaving group-based glycosylation method.

Recently, Liu et al. explored the use of *ortho*-dihydroxyboryl-substituted benzyl thioglycosides as a delivery method for the leaving group-based intramolecular glycosylation [129]. They hypothesized that if boronic acid-derived donor **100** is activated in the presence of glycosyl acceptor **101,** the boronic ester **102** would form as the key intermediate. Upon dissociation of the anomeric C–S bond of the sulfonium intermediate **102**, an oxygen nucleophile on the boronate ester would attack the C-1 center on the opposite side resulting in **103** with good stereoselection (Scheme 23). Initial trials with 3-methylbenzyl alcohol showed good selectivity (α/β = 4.8:1) when boronic acid and NBS were employed. Control experiments with a thiophenyl or a thiobenzyl leaving group showed lower stereoselectivities and a slight reduction in yields. The addition of triflic acid or silver triflate resulted in a significant reduction of stereoselectivity, so further trials were done in the absence of metal or acid reagents. Surprisingly, when IBr was used as a promoter the selectivity reversed resulting in the formation of glycoside **103** in 65% yield and high β-stereoselectivity (α/β = 1:10). The selectivity also reverses when the reaction is carried out in the presence of a coordinating solvent, for example, a similar reaction performed in acetonitrile delivers glycoside **103** in 51% yield (α/β = 1:4). When using less than three equivalents of acceptor to donor ratio, the yield drastically drops giving evidence that the borate intermediate plays an important role in the stereoselection.

## Conclusion

Intramolecular glycosylation has seen dramatic advancements in the past two decades. New tethers, templates and conditions have advanced the synthesis of challenging glycosidic linkages. A more streamlined synthesis of starting materials has also made these methodologies more attractive for use in more complicated multistep syntheses. Despite the advancements made, there are still no definitive rules on why small changes may affect the stereochemical outcomes so dramatically. There is a greater need to study the underlying concepts and rules governing the use of tethers and templates and how to apply them to new systems and targets.
